# Design of Iron-Based Multifunctional Alloys Electrodeposited from Complexing Electrolytes

**DOI:** 10.3390/ma18020263

**Published:** 2025-01-09

**Authors:** Natalia Tsyntsaru, Henrikas Cesiulis, Oksana Bersirova

**Affiliations:** 1 Faculty of Chemistry and Geosciences, Vilnius University, 03225 Vilnius, Lithuania; oksana.bersirova@chgf.vu.lt; 2 Institute of Applied Physics, Moldova State University, 2028 Chisinau, Moldova

**Keywords:** iron alloys, electrodeposition, complexing electrolytes, refractory metals alloys, advanced properties

## Abstract

There is a growing focus on sustainability, characterized by making changes that anticipate future needs and adapting them to present requirements. Sustainability is reflected in various areas of materials science as well. Thus, more research is focused on the fabrication of advanced materials based on earth-abundant metals. The role of iron and its alloys is particularly significant as iron is the second most abundant metal on our planet. Additionally, the electrochemical method offers an environmentally friendly approach for synthesizing multifunctional alloys. Thus, iron can be successfully codeposited with a targeted metal from complexing electrolytes, opening a large horizon for a smart tuning of properties and enabling various applications. In this review, we discuss the practical aspects of the electrodeposition of iron-based alloys from complexing electrolytes, with a focus on refractory metals as multifunctional materials having magnetic, catalytic, mechanical, and antimicrobial/antibacterial properties with advanced thermal, wear, and corrosion resistance. Peculiarities of electrodeposition from complexing electrolytes are practically significant as they can greatly influence the final structure, composition, and designed properties by adjusting the electroactive complexes in the solution. Moreover, these alloys can be further upgraded into composites, multi-layered, hybrid/recovered materials, or high-entropy alloys.

## 1. Introduction: Iron-Based Alloys and Materials for Unlocking Sustainable Versatile Applications

Nowadays, there has been growing consideration on sustainability, characterized by viable changes that enable foreseeing future needs and adjusting them to the present necessities [1]. Sustainability aspects are rising in different branches of materials science as well. Thus, more research is focused on the fabrication of advanced materials based on earth-abundant metals (iron, nickel, and cobalt), especially for catalytic applications [2,3]. Moreover, the role of iron and its compounds is significant because iron is the second most earth-abundant metal. They can be used in various applications, which will be discussed below, but certainly, the magnetic [4] and catalytic [5,6] properties of iron-based advanced materials are acknowledged foremost.

Furthermore, iron-based materials with high theoretical capacity and good mechanical and thermal stability have attracted research interest as electrode materials for alkali metal-ion batteries (AMIBs). Advances in the chemical synthesis and structural design of iron oxide-containing materials for these purposes are discussed elsewhere [7]. In addition, other materials like the FeCo alloy have found applications in lithium–sulfur batteries (LiSBs). Thus, it was confirmed that the FeCo alloy produced by the melt-diffusion method enhances the redox kinetics of polysulfide conversion. In addition to its strong adsorption capabilities for lithium polysulfides (LiPSs), this alloy effectively restricts the migration of LiPSs and significantly reduces their accumulation [8].

Another pertinent field of iron-based materials applicability is linked to their proven biocompatibility and mechanical properties. Thus, iron and iron-based alloys are excellent source materials for clinical cardiac and vascular applications as biodegradable stents [9,10]. However, its relatively low degradation rate limits its use for the healing and remodeling of diseased blood vessels. To address these issues, a multi-purpose fabrication process was employed to develop a bilayer material composed of electroformed iron and iron–phosphorus alloy [11]. Fe-P has good biocompatibility and a favorable effect on the iron degradation rate [12]. Fe–Mn–Pd alloys also reveal a degradation resistance that is one order of magnitude lower than that observed for pure iron [13]. In addition, alloys possessing antimicrobial/antibacterial properties such as Fe-Ag and Fe-Au have been a point of interest for the research community [14,15,16].

The synthesis of iron-based materials and alloys can be realized by various solid-state techniques, among which electrodeposition is often considered a smart alternative. Indeed, electrodeposition is a well-accepted surface modification method that improves the decorative and functional characteristics of a wide variety of materials. Thus, the alternation of variables such as electrolyte composition, pH, temperature, agitation, current density, and potential [17,18,19,20,21,22,23], as well as the application of a magnetic field [24], can tune the composition, microstructure, and interrelated properties of the deposited materials in a wide range.

Furthermore, electrodeposition provides a significant advantage over other fabrication methods for synthesizing micro- and nanomaterials, and various aspects of this technique can be explored further in other studies [25]. It enables the production of materials and small devices with complex geometries that do not require additional processing, resulting in an effective device that is nearly “ready to use”. Another peculiarity discussed in detail in the next section is linked to the electrodeposition of materials from complexing electrolytes. Complexing (chelating) is a powerful tool that can alter the electrode potentials of the metals in the electrolyte by converting the simple ions of more noble metals into complex ions with lower equilibrium potentials. Therefore, it is possible to codeposit metals possessing different standard reduction potentials because the negative shift of equilibrium potential due to the formation of complexes would decrease the exchange current density and improve the deposited layer’s uniformity [26].

The electrodeposition of several types of iron alloys has been under significant attention in recent decades for magnetic applications. Alloys of iron with nickel and cobalt are most frequently used for these purposes [27]. In addition, electroplating methods for depositing industrially valuable magnetic alloys such as Fe-Ni, Fe-Zn, Fe-Co [27,28], and Fe-Pt were investigated [29]. Moreover, Fe-Pd alloys possessing high perpendicular magnetic anisotropy and ferromagnetic shape memory [30,31], Fe-Ga magnetostrictive alloys [32,33], and soft magnetic alloys like Fe-W, Fe-Mo, Fe-Co-W (Mo), and Ni-Fe-W (see for ex.: Refs. [34,35,36,37,38,39,40]) were also studied.

Nonetheless, according to the European Chemicals Agency, nickel and Co(II) salts are substances of high concern [41]. In this view, electrodeposited Fe-Sn alloys were proposed as an alternative to conventional nickel- or cobalt-based magnetic materials [42,43]. In this case, the intermetallic compounds are formed, and ferromagnetic iron-rich alloys of the compositions Fe_3_Sn_2_, Fe_5_Sn_3_, and Fe_3_Sn are of interest to industrial applications due to their affordable cost and sustainability aspects [44]. Other magnetic iron alloys, such as Fe-P alloys [45,46,47,48] and less common Fe-Re alloys [49], were also investigated.

The advancements in green chemistry and engineering are driving toward the implementation of new processes or redesigning existing ones. The development of 3d transition metal-based catalysts, particularly based on earth-abundant metals like iron, has been scientifically proven over decades and is even more important now [50,51]. Due to their adjustable chemical reactivity, catalytic efficiency, and corrosion resistance [52], iron-based catalysts have been widely studied for various applications, including water purification (e.g., Fe-Mn alloys and electrodeposited zero-valent iron for Fenton reaction [53,54,55]), CO_2_ removal (e.g., Fe-Ni nanoalloys [56]), and oxygen reduction (e.g., Fe-Co-Pt alloys [57]).

When considering corrosion properties, fabricating composite materials is one of the common approaches. This approach allows for the combination of the advantageous properties of the second-phase particles within the metallic matrix, resulting in novel materials possessing tailored characteristics. Composite coatings with alumina particles have been the most extensively studied among particle-reinforced composites. Thus, electrodeposited composite iron-based coatings such as Fe-W/Al_2_O_3_ [58,59] or Ni-Fe/Al_2_O_3_ [60] have been studied and evaluated as an effective sustainable alternative to electrodeposited hard chromium coatings. Also, Ni-Fe/WC composites have improved resistance to corrosion in comparison with their Ni-Fe counterparts [61].

One of the main peculiarities of alloys obtained by electrodeposition is the formation of solid solution phases of metals, which often cannot be predicted from a phase diagram, i.e., the formation of oversaturated solutions. Thus, during electrodeposition, the thermodynamically unstable structures of alloys are forming. The structural relaxation and recrystallization processes after annealing lead to changes in the grain size, microstructure, and properties of electrodeposited alloys. In the case of iron-based advanced materials, the recrystallization temperature is rather high, typically ranging from 400 to 600 °C [62,63,64,65]. The accurate control of heating temperature allows for tuning of the alloy’s properties, making these materials suitable for thermal-resistant applications.

Though several review articles have been published on different aspects of iron-group materials, the present review aims to discuss the peculiarities and possibilities of the electrochemical design of iron-containing alloys from complexing electrolytes, the advanced properties associated with these alloys, and the perspectives on the future development of the electrodeposited materials.

## 2. Codeposition of Metals and Peculiarities of Iron-Based Alloys Electrodeposition from Complexing Electrolytes

The thermodynamic conditions for the alloy electrodeposition are quite well understood from the macroscopic standpoint. This approach is comprehensively discussed in [22,26,66,67,68]. The key point in the macroscopic thermodynamic description of alloy electrodeposition is an analysis of the Nernst equation written for the equilibria between metal ions in the solution and metal (origin or foreign). Due to the activities of elements in the alloy differing from the unit, the Nernst equations for the two equilibria alloy/solution of the electroreduction of a binary (For simplicity, the binary alloys are described. However, the same approach can be applied to the multicomponent alloys (ternary, quarterly, etc.)) alloy for metals *A* and *B* can be presented as:

(1) $$\begin{array}{c} A^{z_{A}+}+z_{A}e^{-}\to A_{\mathrm{alloy}}\\ B^{z_{B}+}+z_{B}e^{-}\to B_{\mathrm{alloy}} \end{array}$$

where the equilibrium potentials $E_{eq,alloy}^{\left(i\right)}$ for each alloying component can be written as follows:

(2) $$\begin{array}{l} E_{eq,alloy}^{\left(A\right)}=E_{A}^{0\prime }+\frac{RT}{z_{A}F}\ln\frac{a_{A^{z_{A}+}}}{a_{A,alloy}}\\ E_{eq,alloy}^{\left(B\right)}=E_{B}^{0\prime }+\frac{RT}{z_{B}F}\ln\frac{a_{B^{z_{B}+}}}{a_{B,alloy}} \end{array}$$

where $E_{A}^{0\prime }$ and $E_{B}^{0\prime }$ are formal potentials of the components mentioned above, *A* and *B*, respectively; and

$a_{A^{z_{A}+}},\;a_{B^{z_{B}+}},\;a_{A,alloy},a_{B,alloy}$ is the activity of ions in the solution and components *A* and *B* in the alloy, respectively. Other marks are standard.

The most favorable conditions for the electrodeposition of an alloy consisting of two metals A and B are usually achieved when the equilibrium potentials of those metals are close. In this case, the equilibrium composition of the alloys can be expressed by equilibration of Equations (1) and (2), and it might be represented as follows:

(3) $$\frac{{a^{z_{A}}}_{A,alloy}}{{a^{z_{B}}}_{B,alloy}}=\frac{{a^{z_{A}}}_{A^{z_{A}+}}}{{a^{z_{B}}}_{B^{z_{B}+}}}\exp\left[\frac{F}{RT}\left(E_{B}^{0\prime }-E_{A}^{0\prime }\right)\right]$$

As seen from Equation (3) (please note that the equation is expressed differently in [26]), the composition of alloys depends on the activities (or concentrations) of metal ions in the solution. Notably, the activity coefficients can be neglected for moderately dilute solutions (up to 1–2 M), and concentrations of components are used instead of ones as they appear in the Nernst equation in the logarithmic form. Thus, for example, if the activity coefficient is only 0.8, instead of an assumed value of 1.0, the resulting error in the value of *E_eq_* is only (5.7/*z*) mV (where “*z*” is the number of electrons transferred in the reaction per molecule). Therefore, in many cases, the concentrations are used instead of activities in the thermodynamic equations [66].

Due to various complexing agents in the solutions, the concentrations of solvated “simple” metal ions decrease, leading to a negative shift in the equilibrium potentials. Here, we should dive into how this negative shift is formed in the complexing electrolytes of a metal (M).

The equilibrium in the solution containing complexes is settled between the free metal ions M^z+^, free ligand L, and various complexes ML_j_^z+^:

(4) $$M^{z+}{+\mathrm{jL}}^{m}\Leftrightarrow {\mathrm{ML}}_{j}^{z+\mathrm{jm}}$$

where j = 1 … n; m is a charge of a ligand.

In the simplest case, when a single complex can be formed in the solution, the equilibria may be represented by the equilibrium constant called cumulative stability constant (β*_n_*), which can be presented as:

(5) $$\beta_{n}=\frac{\left[{\mathrm{ML}}_{n}^{z+\mathrm{nm}}\right]}{\left[M^{z+}\right]{\left[L^{m}\right]}^{j}}$$

If stable complexes are formed, the value β*_n_* >> 1. Usually, the concentration of ligands in the solution is higher than the total metal concentration. Therefore, it essentially reduces the concentration of free metal ions in the solution

(6) $$\left[M^{z+}\right]=\frac{1}{\beta_{n}}\cdot \frac{\left[{\mathrm{ML}}_{n}^{z+\mathrm{nm}}\right]}{{\left[L^{m}\right]}^{j}}$$

and causes an essential negative shift in equilibrium (thermodynamic) potential

(7) $$E_{eq}^{\left(M\right)}=E_{M}^{0\prime }+\frac{RT}{zF}\ln\left(\frac{\left[{\mathrm{ML}}_{n}^{z+\mathrm{nm}}\right]}{{\left[L^{m}\right]}^{j}}\right)-\frac{RT}{zF}\ln\beta_{n}$$

Consequently, it also causes the negative shift of the metal deposition potential, *E_dep_*, to more negative values:

(8) $$E_{dep}^{\left(M\right)}=E_{M}^{0\prime }+\frac{RT}{zF}\ln\left(\frac{\left[{\mathrm{ML}}_{n}^{z+\mathrm{nm}}\right]}{{\left[L^{m}\right]}^{j}}\right)-\frac{RT}{zF}\ln\beta_{n}+\eta$$

The term η in Equation (8) represents the difference between deposition and equilibrium potentials:

(9) $$\eta =E_{dep}^{\left(M\right)}-E_{eq}^{\left(M\right)}$$

Understanding the possibilities of alloy electrodeposition is crucial. However, predicting the composition of electrodeposited alloys based solely on thermodynamic data is quite complicated due to the kinetic peculiarities of metal electroreduction. The complexing electrolytes for metals and alloys electrodeposition are essential because the formation of complexes decreases the exchange current density (η increases) and could lead to the improved uniformity, smoothness, and brightness of the deposits [66].

On the other hand, to successfully investigate the electrochemical processes at the metal/solution interphase in the presence of complexing agents, attention should also be paid to the solution chemistry. In other words, evaluating the distribution of complexes and ligands in the solution volume is necessary. For this purpose, it is possible to extract concentrations of all species formed in the solution after the dissolution of corresponding salts by solving the system of equations using commercially available software, e.g., Maple6, MathCad, MINDTEQ, Meduse, or others. The set of the system of equations has to include the following relations and quantities:

(i) The equilibrium constants for all compounds added to or formed in the solutions: acids deprotonation, hydrolysis, polymerization, stability constants of metal complexes with ligands, etc.;
(ii) The mass balance equations ${\left[J\right]}_{tot}=\sum \left[J_{i}^{n+/-}\right]$ for all forms in the equilibrium mixture, and
(iii) The charge balance “Cat” and “An” denote cation and anion, respectively.

Certainly, the calculated distributions of species in the electrolyte can vary depending on the concentration of precursors and pH, leading to changes in the deposit composition and properties, respectively. For example, when assessing the species distribution in citrate- and tungstate-based electrolytes, 20 to 32 different species can be identified and included in the calculations.

**Figure 1:**
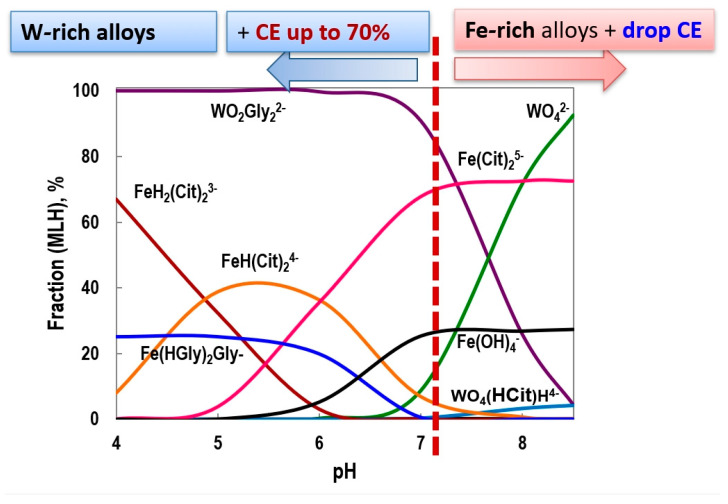
Calculated distribution of various complexes in the glycolate-citrate electrolyte vs. pH (based on the data reported in [69]) and variation of tungsten/iron content and current efficiency of Fe-W electrodeposits. WO_4_^2−^, Fe(Cit)_2_^5−^, Fe(OH)_4_^2−^, WO_4_(HCit)H^4−^, WO_2_Gly_2_^2−^, FeH_2_(Cit)_2_^3−^, FeH(Cit)_2_^4−^, Fe(HGly)_2_Gly^−^ are the species formed in the glycolate-citrate solutions at Fe-W alloy electrodeposition. The distribution of species is represented by the colorful lines with the tag of the corresponding species next to it. The red dashed line corresponds to the transition from W-rich to Fe-rich alloys.

This approach has proven effective in:

(i) The formulation of a thermodynamically stable solution for the electrodeposition of Fe-rich or W-rich alloys from the Fe(III)-based glycolate-citrate electrolytes (Figure 1). Additionally, the distribution of electrochemically active complexes varies with pH, which in turn affects current efficiency (CE) and the properties of the resulting materials [69,70];
(ii) Understanding the correlation between W content in the Co-W alloys as a function of pH and various citrate complexes of WO_4_^2−^ [34];
(iii) Determination of conditions that allowed for the increase in the deposition rate of Ni-W alloys [71];
(iv) Impeding the deposition of Ni during Ni-W alloy electrodeposition processes [66];

Furthermore, the evaluation of species distribution in the complexing electrolytes allowed authors to clarify the following:

(v) The pH range for stable ferric-stannous tartrate-chloride-sulfate electrolytes for Fe-Sn electrodeposition [42];
(vi) The role of glycine in the Fe-P alloy electrodeposition [72];
(vii) Codeposition conditions, e.g., for CoFeNiCu alloys electrodeposition from citrate solutions [73];
(viii) Pourbaix diagrams for systems containing few metals and complexing agents, e.g., for Cu-Co-Fe in acetate solutions [74] and for Fe-V-O-H metastable materials [75].

It is important to acknowledge that complexes significantly influence the structure, composition, and properties of materials. This is due to their direct involvement in forming electroactive species either within the bulk material or on the surface of the electrodes during the deposition process. Thus, the well-known peculiarity associated with the electrodeposition of materials containing iron group metals with refractory metals mentioned above is linked to the mechanism of codeposition of such materials, which is classified as induced codeposition [76,77]. This is unlike anomalous deposition [78], which is a common case for the codeposition of iron-group metals (e.g., FeNi alloys) or the codeposition of Fe with Zn or Cd.

The understanding of the induced codeposition mechanism and the role of complexes in the case of refractory metals codeposition was overviewed elsewhere [34,66]. We will not enter into a deep discussion on this matter here, but it can only be specified that either “bulk” and/or “adsorbed” complexes formed during induced codeposition could be correlated to the experimentally observed results.

However, while developing iron-based materials with rhenium under an induced codeposition mechanism, some particularities should be considered: namely, perrhenate ions (the source of rhenium) do not form polynuclear complexes in acidic solutions in contrast to WO_4_^2−^. Therefore, rhenium codeposition with iron group metals is possible from weakly acidic or neutral solutions, preferably using citrate as a complexing agent. The compositions of electrolytes, electrodeposition conditions, compositions of obtained alloys, and their properties are reviewed in [66,79,80].

Also, it should be considered that the Nernst potential (E_o_^red^) of Re in the perrhenate ions solutions depends on the pH. It is +0.363 V at pH 0, −0.110 V at pH 7, and –0.584 V at pH 14.0. These values are more positive than those for the iron group metals, e.g., E_o_^red^ for Fe^2+^/Fe and Fe^3+^/Fe are −0.447 V and −0.037 V, respectively [81].

However, the electroreduction of ReO_4_^−^ is highly irreversible. The ReO_4_^−^ reduction wave occurs just before the onset of electrolyte decomposition, but the electroreduction in weakly acidic citrate or oxalate solutions is facile, resulting in a polarographic wave at much more positive potentials [82]. It is explained by the formation of electrochemically active (ReO_4_·H_2_Cit)^2−^ complexes at pH above 3. The role of this complex in the codeposition mechanism of Re with Fe has been discussed in [66,80]. However, based on the UV–vis absorption spectroscopy data obtained in the citrate-perrhenate system at pH 5, it is indicated that the ReO^4−^ ions are unlikely to chemically bind to the dissociated citric acid. Therefore, it is assumed that even in the presence of citrate ions, the ReO_4_^−^ ion remains hydrated [83]. In that respect, it is an interesting consideration that the overall reaction leading to the formation of rhenium is influenced by the presence of H^+^ ions, which depend on the adsorption process and therefore on the electrode surface nature. This means that the surfaces with high hydrogen adsorption could facilitate the reduction of perrhenate ions to Re [84].

Moreover, to explain some experimental results of induced codeposition, a new theoretical approach was developed that assumes the formation of an N-dimensional fractal cluster in the solution [85]. This theory enables us to avoid the difficulties of the classical theory of nucleation operating with the concepts of surface energy, which is intrinsic to a geometrical surface. In this case, the electrochemical kinetics of nucleation rather than electrochemical reaction is considered. In this sense, the nucleation stage should be considered as the step following the formation of an ad-particle on the surface.

By applying a non-linear approximation of nanonucleation from fractal solutions for the interpretation of induced codeposition of iron group alloys with refractory metals, it was possible to explain that a high rate of propagation of crystallization front had influence on:

(i) Nanocrystallinity of the electrodeposited new phases;
(ii) Formation of composites containing oxide-hydroxide inclusions;
(iii) Absorbed hydrogen at high current density.

Another peculiarity that should be taken into account in the developed electrolytes is the stability of Fe(II) species [86] because it can often hinder the usage of iron-based materials for scale-up applications. Electrolytes based on Fe(II) salts are unstable thermodynamically, and the solution content is governed by the Fe(II) oxidation kinetics [27].

It was shown that citrates and ammonia [27] inhibit the kinetics of Fe(II) oxidation to Fe(III) but cannot prevent it. Some attempts were made to prolong bath life by adding reducing agents, such as L-ascorbic acid [86,87], but those compounds can affect the bath maintenance and coatings properties. In the case of Fe-Ag alloy electrodeposition, an instability caused by the reduction of Ag(I) by Fe(II) was partially resolved by adding 1% Fe^3+^ [16]. The solution containing Fe(II) will oxidize gradually unless used regularly. The time and efforts required to restore the electrolyte to an operable condition may outweigh the economic benefit of depositing a lower-cost material; e.g., it is necessary to reduce the Fe(III) ion before using a freshly prepared bath, and this process might require 24–48 h [27]. Therefore, the electrodeposition of Fe alloys from the solutions based on Fe(III) compounds was also investigated for the fabrication of Fe-W, Fe-Ni, Fe-Pt, and ternary alloys [69,88,89,90,91].

## 3. Versatile Materials Based on Iron-Based Alloys

In this section, the main focus will be on the practical aspects of electrodeposited iron-based alloys containing refractory metals, primarily tungsten-based alloys, as these alloys can be adapted for various applications.

From an application point of view, iron-based alloys with refractory metals are an important class of functional materials that are characterized by a distinctive set of properties linked to the presence of high melting refractory metal, such as increased heat resistance and stability at high temperatures, high corrosion resistance, and excellent mechanical properties [34,92,93,94]. These alloys are advanced materials for strategic components in many industries (hard coatings, catalysts, aerospace, chemical, nuclear, defense, biomedical, etc.), including as a component of superalloys [95]. The codeposition of refractory metals with iron group metals leads to the obtainment of high-quality electrodeposits with higher current efficiencies [63,69,96], and in many cases, they can replace high-cost materials. However, the research on iron alloys is still growing compared with other metals due to the sustainability aspects and advanced properties of iron-based materials.

### 3.1. Structural Peculiarities, Mechanical, Thermal Resistance, and Magnetic Properties

#### 3.1.1. Structural Peculiarities and Mechanical Properties

The key factors affecting the properties of the electrodeposited material are directly linked to its structure and chemical composition. The electrochemical approach induces the precise scenario for an alloy synthesis with targeted amorphous and/or nano-structure, composition, internal ordering, and texture. Thus, the main characteristic of electrodeposited iron-based materials with refractory metals is their nanocrystalline structure, which primarily impacts those alloys’ hardness and is correlated to alloy composition in the electrodeposit and reduced crystallite size [36,38,58,63]. According to the Hall–Petch relation, the mechanical properties typically improve as the grain size decreases due to the increase in grain boundaries, which hinders dislocation motion. Moreover, effects such as nanostructuring and solid solution strengthening will also be involved.

However, there is a critical value of crystallite size at which the Hall–Petch relation will be distorted. In the case of Co-W [97] and Ni-W [98] alloys, the breakdown occurs at crystallite sizes of 5 nm and 10 nm, respectively. Notably, the same tendency was also observed in the case of more complex systems such as, e.g., high-entropy NiFeCo-W alloys [99]. Remarkably, it often corresponds to the transition to the amorphous-like or ultra-nanocrystalline structure of electrodeposited materials [100], which promotes the diverging of the shear bands that lead to the nanohardness decrease. However, the ultra-nanocrystalline structure brings other benefits, e.g., it can increase catalytic properties, which will be discussed later below.

**Figure 2:**
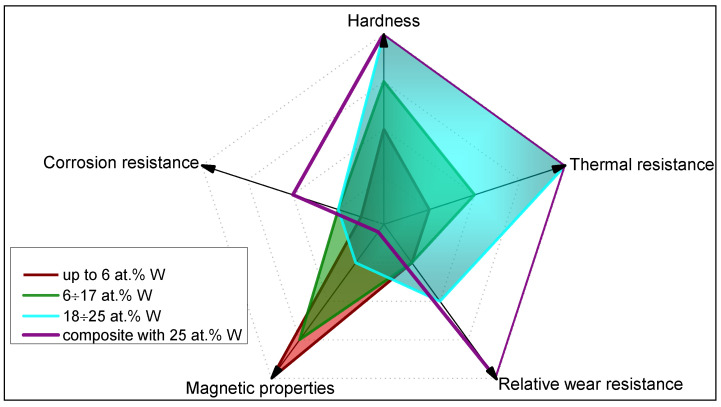
Properties mapping of electrodeposited Fe-W alloys as a function of tungsten content in the coating. In addition, properties of Fe-W/Al_2_O_3_ composited coating are presented for comparison. The black arrows show the increase in the corresponding characteristics (a.u.). The Fe-W alloys were electrodeposited from environmentally sustainable glycolate-citrate electrolytes [69].

The hardness of Fe-W alloys increases from 4.1 GPa for the Fe-6 at.% W alloy to 10.4 GPa for the Fe-25 at.% W alloy. Along with this, the elastic modulus also rises significantly, increasing from 83 GPa to 216 GPa. This trend is strongly associated with the development of a stable intermetallic Fe_2_W phase in alloys that contain more than 16 at.% of tungsten. [36]. As a result, smart tuning of mechanical properties can be accomplished by varying the alloy composition and applied experimental conditions (Figure 2). Concerning alloy composition, it can be underlined that under similar testing conditions and refractory metal content, generally, iron-based alloys have higher hardness than cobalt- or nickel-based electrodeposited materials [63], which encourages the use of iron-based materials for hard coating applications.

On the other hand, experimental conditions such as volume current density (VCD) can also affect drastically the hardness and even corrosion, as demonstrated in [101,102,103,104]. The VCD describes the ratio of current to the volume of an electrolyte (I/V). It is an important characteristic, especially taking into account the upscaling of lab processes, because usually only common characteristics such as pH, composition, temperature, current density, or potential are considered. The dependence of alloy composition on VCD is linked to a different rate of the metal complex (gluconate, citrate, etc.) concentration change during induced codeposition. VCD is mainly specific to the induced codeposition of tungsten alloys, and it does not influence the electrodeposition of industrially important metals such as nickel or chromium from non-complexing electrolytes [101].

During the codeposition of refractory metals, significant differences in composition, morphology, and microhardness may occur depending on the electrolyte volume at either a constant electrode potential or current density [102]. Thus, depending on the VCD, electrodeposited nanocrystalline alloys contain not only iron group and refractory metals but also different amounts of intercalated oxides and hydroxides, and even alloyed hydrogen (e.g., Fe-W-H) [102,105], that will ultimately have an impact on the properties. Another important experimental parameter is the nature of the counter electrode (material from which the anode is created from and whether it is insoluble or soluble). It affects the intrinsic and extrinsic characteristics of refractory metal alloys during induced codeposition from complexing electrolytes, including the rate of deposition and hardness [103,106].

#### 3.1.2. Thermal Resistance

**Figure 3:**
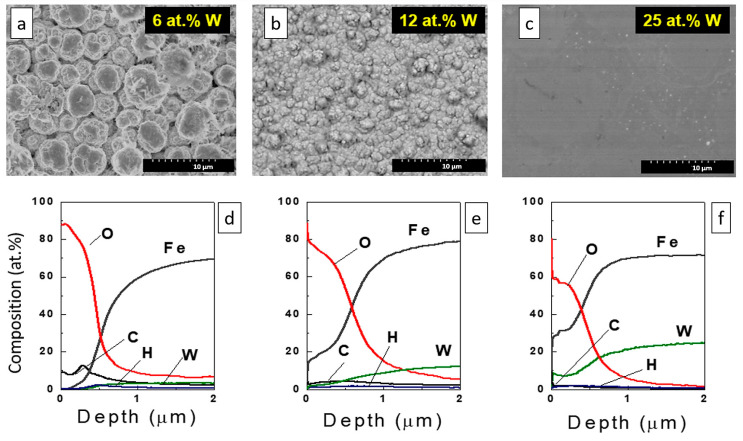
The representative SEM images of electrodeposited Fe-W alloys and the distribution of elements in the top layers of the 10 µm thick coatings. SEM images and the corresponding distribution of elements: (**a**,**d**) 6 at.% of W, (**b**,**e**) 12 at.% of W, (**c**,**f**)—25 6 at.% of W. Inspired from [109].

The presence of inclusions and abundant evolution of hydrogen during electrodeposition can promote the brittleness of the obtained iron-based materials [93]. In this view, annealing can decrease the internal stress of the deposits and advance the mechanical properties. During annealing, electrodeposited refractory alloys will recrystallize, forming coarser grains and new phases (oxides, intermetallic phases, carbides, etc.) [62,63,64,70,107,108]. Typically, refractory metals-rich alloys, in comparison with alloys having higher iron content (Figure 3a–c), show improved thermal resistance linked to the formation of thermodynamically stable nanostructures with decreased energies of segregation and grain boundaries.

For instance, the XRD amorphous-like structure of Fe-W alloys can withstand temperatures of up to 600 °C, which is higher than that for Ni-W or Co-W alloys with the same tungsten content [63,70]. Above this temperature, a partial recrystallization occurs in the Fe-W alloys, but some amorphous peaks are still presented in XRD patterns even at 800 °C [109]. The nanocrystalline peaks of annealed alloys usually are attributed to α-Fe, Fe_2_W, FeWO_4_, and Fe_6_W_6_C phases [109,110], although the Fe_7_W_6_ phase [111] can also be present in the as-deposited coating, having a more coarse-grained structure and lower tungsten content. However, tungsten-rich alloys, electrodeposited from citrate-based electrolytes and annealed at 700 °C [110] or 800 °C [109], contain uncommon phases such as Fe_6_W_6_C and Fe_3_W_3_C.

The appearance of hydrogen-, carbon-, and oxygen-containing phases is practically independent of W content (Figure 3d–f), and it is attributed mostly to the deposition from complexing electrolytes having organic ligands, which afterwards affect the microstructure and properties of as-deposited and annealed alloys [109,110,112,113]. Moreover, the light elements are typically present in the top layers of the deposits, and their contents essentially decrease when moving deeper from the surface up to 2 μm (see Figure 3). This effect does not depend on the content of W in the alloys or the morphology of the surface. The ratio of content W to Fe remains almost constant along the entire thickness of electrodeposits.

Moreover, the dependence of annealed alloy properties (hardness, wear, and corrosion resistance) on temperature usually will have an optimum. This optimum for heat treatment will depend on the initial tungsten content in the coating and the “amorphous state” of as-deposited Fe-W coatings, which is connected to a shift in the annealing temperature from 500 °C [111] to 600 °C [109] and 700 °C [110]. Also, it is connected to the precipitation and dispersion of fine α-Fe crystallite and Fe_3_W_3_C particles in the Fe-W alloys [114]. Thus, for the as-deposited coatings having 25 ÷ 36 at.% of W and annealed ones, this leads to an increase in hardness ranging from ~8 ÷ 10 GPa to ~16 ÷ 20 GPa. At higher temperatures than 800 °C, coarse grains are formed, which negatively impacts the hardness of the material [109,113]. Overall, we should emphasize that the increased content of refractory metal in iron alloys will facilitate a transition to a more thermal-resistant state of deposited materials (Figure 2).

#### 3.1.3. Magnetic Properties

Electrodeposited Fe-based alloys are extensively studied for applications in magnetic devices because it is possible to tune both the composition and structure of obtained materials. To achieve the optimal soft magnetic properties, grain size control is critical. According to a model proposed by Herzer [115], coercivity *H_c_* is proportional to the sixth power of average grain size, i.e., the so-called “*D6*” law. As was shown in [116], for Co-Fe alloys electrodeposited from citrate-based baths, the average grain sizes in deposits are around 10–20 nm, which is quite fine and beneficial for achieving optimal soft magnetic properties. So, the CoFe films electrodeposited from citrate-added baths exhibited better soft magnetic properties than those deposited from citrate-free baths under the applied experimental conditions. A similar behavior was obtained for Ni_86.0_Fe_9.8_W_1.3_Cu_2.9_ alloy electrodeposited from citrate baths: during annealing, the mean crystallite size increased from 12 to 29 nm, resulting in reduced magnetization and broadened magnetic hysteresis and thus increased loss in the active power and decreased strength of the coercive field [117]. CoFeNi thick films electrodeposited from citrate baths also obey a “*D6*” law for the correlation between crystallite size and coercivity, but the saturation magnetization is not influenced by the crystallite size and depends on the chemical composition [118]. However, in the case of electrodeposited Fe-Pd alloys from citrate-ammonia baths, deposited alloys are almost disordered, whereas annealing at 400–600 °C promotes crystallinity, and the magnetic coercivity increases up to 10 times [119].

Exploration of soft magnetic iron-based alloys such as NiFeCo can be further extended by the incorporation of rare earth elements (e.g., Tb and Dy), which contribute to the giant magnetostriction of electrodeposited materials that can be engaged in resonant frequency sensing devices [120]. In addition, a magnetic field applied during the electrodeposition can provide a feasible way to produce oriented magnetostrictive CoFe materials for dual-mode flexible sensors [121].

In the case of iron-based materials with tungsten, the magnetic properties are strongly dependent on the electrochemical design. Thus, the experimental conditions (pH, temperature, current/potential) will affect the structure, composition and, in the end, the transition to a semi-hard or soft magnetic nature of deposited materials. Thus, hard and soft magnetic properties of refractory binary [97,122,123] and ternary [40,124,125,126] alloys and more complex systems [127] with iron have been reported. The hard and soft magnetic properties are more likely to be found in cobalt-based alloys [97], while iron-based alloys with refractory metals are generally characterized by a low coercive field (*Hc* < 200 Oe). Therefore, soft or semi-soft magnetic properties could be expected, which can be applied to sensors, read/write heads in hard discs, and microelectromechanical systems.

Here, we should underline that, commonly with an increase in the non-magnetic phase (refractory metals in our case), the saturation magnetization will decrease. Thus, Fe-W alloys electrodeposited at room temperature display a much sharper decrease in saturation magnetization for deposits having 12 at.% of W, but deposition at 65 °C (16 at.% of W) leads to the retention of rather high magnetization [36]. This behavior is attributed to the thermodynamic constraints of nucleation and crystallite phase formation from complexing electrolytes at room and elevated bath temperatures. An amorphous-like structure can be formed at room temperature at a lower content of refractory metals (12–14 at.% of W). However, at higher temperatures, the XRD amorphous peaks start to appear only at 16–18 at.% of W.

Furthermore, the nanocrystalline deposits have a higher value of the saturation magnetization caused by the presence of the α-Fe phase in the Fe-W alloy, while XRD amorphous phases such as solid solution of tungsten in iron, W(Fe), and intermetallic Fe_2_W contribute to a decrease in saturation [36]. The coercivity evaluation of such materials illustrates an overall semi-soft magnetic character with values in the range of 10 ÷ 190 Oe depending on tungsten content (Figure 2).

### 3.2. Wear and Corrosion Resistance

One of the important aspects of research on the iron group metals refractory alloys is connected to finding materials that can replace electrodeposited chromium, whose synthesis involves restricted compounds of Cr(VI) [128]. Hence, iron-base alloys are an important pillar that addresses sustainable issues linked to Cr(VI) and the subsequent use of chromium as a hard and corrosion-resistant coating. Additionally, our group’s research indicates that the wear rate of tungsten-based alloys under identical testing conditions is comparable with that of electrodeposited chromium [34]. Other researchers obtained similar data using different test setups [129]. In general, comparing the tribological behavior of different materials is quite complicated due to the involvement of different tribo-systems and test conditions. Usually, the evaluation of tribological properties is mostly linked to material characteristics, namely the ratio between hardness (or plasticity) and wear [98,130]. The higher ratio between the hardness and elastic modulus (elastic strain to failure) provides a material with enhanced resistance to permanent plastic deformation, which could diminish wear caused by this deformation [130]. Indeed, the elastic-plastic behavior was the prevailing cause of wear under dry friction conditions of electrodeposited Ni-W alloys [98,131]. Moreover, the formation of a stable hexagonal close-packed (hcp) structure that impedes the plastic flow in Co-W electrodeposits led to an improved wear resistance at high applied loads [34,132].

Nevertheless, the tribological behavior of Fe-W alloys under dry friction conditions is primarily influenced not only by their mechanical properties but also by the chemical stability of iron during fretting, which involves the tribo-oxidation of the alloys [133]. Iron oxide particles that form in the wear track act as an abrasive third body, which increases the asperity contact between the material and counter-body, thus resulting in a high coefficient of friction and larger wear volume than in the case of Ni- or Co-based alloys. It should be taken into account that increased refractory metal content (low iron content) in the deposits and heat treatment of as-deposited alloys will increase the relative wear and corrosion resistance of iron-based materials [66,98,133].

Another possibility to improve the wear and corrosion resistances of iron-based materials like Fe-W is to introduce a new alloying element, e.g., Zn, P, and La, into binary alloys. Although the tribological and corrosion properties usually improve with the increasing content of newly alloyed components, this approach will provide only a temporary remedy until iron particles start to oxidize [134,135,136].

However, using an environmentally sustainable thin layer (1 µm) of rapeseed oil significantly reduced the tribo-oxidation and wear of Fe-W alloys under lubricating conditions [137]. Thus, the particles of iron oxide were absent in the wear track and at the surface of the counter-body, and the roughness inside the wear track was very close to the initial surface roughness. Hence, the lubricating conditions can considerably improve the tribological characteristics of Fe-W coatings. Nevertheless, the liquid lubricant in this case must be injected regularly to extend the lifetime of the coating, which can only apply to some specific devices.

In this view, composites containing conductive or non-conducting compounds provide a larger stage for innovative solutions [60]. Moreover, composites combine the properties of an alloy with the chemical and physical properties of second-phase particles, thus producing a material with advanced characteristics compared with its analog having the same composition (Figure 2) [58,59]. Usually, it is possible to increase the hardness of the composites, which is positively affected by grain refining, dispersion strengthening, and the texturing effect induced by particles. In addition, due to the self-lubricating effect, the wear resistance can also be improved by choosing the appropriate composites based on a metal matrix and particles such as ZrO_2_, SiC, or Al_2_O_3_ [138].

It is important to note that introducing particles does not guarantee that the resulting composite will have improved characteristics. Frequently, it can lead to increased porosity, low adhesion to the metallic matrix, and peculiarities of codeposition, which could increase, for example, the corrosion rate. Thus, the Al_2_O_3_ particles in the Ni-Fe/Al_2_O_3_ composite had an overall unfavorable influence on the corrosion resistance due to the increased porosity, roughness, and inhomogeneity of the deposits [60]. Therefore, smart design of composites is needed.

Another approach involving a trilaminar structured composite has been applied to protect the Fe-W alloy. Namely, a silane cross-linked graphene oxide layer and hydrophobic organosilane layer were formed on top of the Fe-W amorphous alloy layer to protect it against corrosion [139]. This approach of adjusting the morphology of the electrodeposited Fe-W alloy layer provided the possibility to decrease the corrosion rate by three orders of magnitude and increase overall hydrophobic performance (the contact angle was 141.7 degrees).

### 3.3. Catalysts Based on Electrodeposited Iron-Based Materials and Upgraded Systems

**Figure 4:**
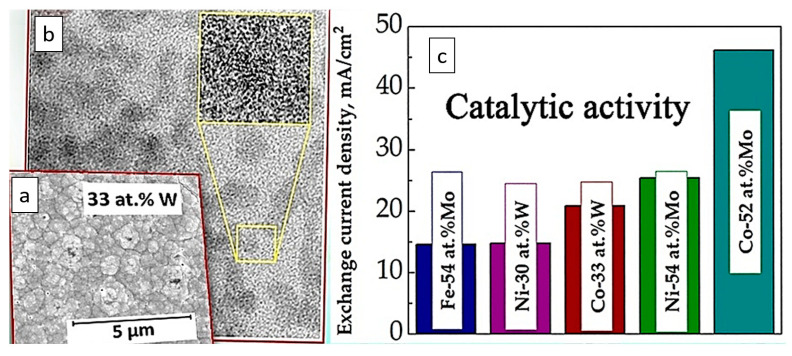
Representative SEM (**a**) and TEM (**b**) images and exchange current densities (**c**) of ultra-nanocrystalline tungsten and molybdenum-rich iron group metal alloys, based on the data [100,155].

Currently, electrodeposited alloys are extensively studied as electrocatalysts for water electrolysis and other chemical reactions. A crucial factor in electrocatalysis is the choice of electrode materials that exhibit high electrocatalytic activity. This quality is essential for reducing the costs of the process. The efficiency depends on two main factors: the composition and structure of electrodeposited material and the electrochemically active area of the catalyst. It was confirmed that a cauliflower-like (Figure 4a), ultra-nanocrystalline structure (crystal grain size below 5 nm) (Figure 4b) of the electrode material leads to an enhancement in the electrocatalytic performance. The fine crystallites of the material increase its surface-over-volume ratio and, thus, a larger surface area is accessible for reactants in the electrochemical reactions [100].

We should highlight an important factor that influences the catalytic activity of electrodeposited coatings, namely the electrochemically active surface area (EASA). By knowing the EASA, it is possible to evaluate the intrinsic catalytic activity of the material and distinguish which of the parameters contributed to the catalytic performance of investigated electrodes, especially in the case of 3D electrodes (electrodeposition on porous electrode, metallic foam).

Electrochemical impedance spectroscopy (EIS) is an effective method for evaluating EASA. Various parameters of EIS data can be used for the EASA estimation depending on the reactions of the catalytic process: (a) capacitance of the double electric layer (reaction involves adsorbed intermediates); (b) Warburg impedance (reaction with limiting mass transfer rate); and (c) charge transfer resistance (reactions with slow charge transfer rate, e.g., HER, OER) [140,141,142]. For instance, by using a 3D electrode (Ni-foam) and co-doping FeW-based catalysts with Ni_3_S_2_/NiS, it was possible to improve the catalytic performance of elaborated materials toward OER and urea oxidation [143] or towards both HER and OER [144].

As discussed earlier, electrodeposited nanocrystalline iron-based coatings with tungsten and other refractory metals are versatile materials that can also be applied in catalysis. The analysis of the electronic configuration of d-metals shows that the codeposition of two or more such metals from two branches of Balandin’s volcano curve could improve and even exceed their individual electrocatalytic properties [145]. Thus, iron-based materials have been employed as catalysts for: methanol and ethanol oxidation [146,147]; hydrogen (HER) and oxygen (OER) evolution reactions [100,148,149]; and photoelectrochemical water splitting by dealloying of the electrodeposited Fe-W alloy [150].

The catalytic activity of iron-based materials with tungsten is closely linked to the composition, structure/phases of the alloys, and pH of the working medium. Moreover, a significant improvement in the catalytic activity leading to a visible reduction in the hydrogen overpotential and an increase in the apparent exchange current density was noticed with the increase in the temperature of the working medium [100]. Generally, the high content of the refractory metal and ultra-nanocrystalline structure (crystallite size below 10 nm) determines the enhanced catalytic properties of such alloys coupled with reasonable corrosion resistance in acidic solutions, which for example are needed for methanol oxidation [100,146,151].

The high catalytic activity of the ultra-nanocrystalline tungsten alloys (containing 30 at.% of W) for HER application can also be ascribed to the formation of stable intermetallic phases [152], ensuring optimal metal distribution over the surface and producing larger catalytically active sites. However, the catalytic activity of CoW-based alloys is usually higher than that of iron-containing analog [100]; see Figure 4c.

A considerable part of research involving iron-based materials is focused on the design of effective catalysts based on Fe binary and ternary alloys with Mo [153,154] because they have the potential to be applied as an efficient HER electrocatalyst in alkaline media. Among effective Mo-rich (52–54 at.%) alloys, the apparent exchange current density of hydrogen evolution for Co-Mo electrodeposits (Figure 4c) was considerably higher than that for Ni–Mo and Fe-Mo coatings [155].

The overvoltage of a material during the hydrogen evolution reaction (HER) at a specific current density is often used as a criterion to assess its catalytic activity. This allows for a comparison of the catalytic performance of different materials for HER and OER. Thus, at room temperature, the overvoltage of HER at 100 mA cm^−2^ (η_100_) in alkaline media for Fe-Mo alloys is −0.22 V [155]; for Fe-W, it is −0.3 V [100]. These values are higher than those obtained for alloys containing only iron-group metals FeCoNi or FeCoNiP, where η_100_ varies from −0.196 V to −0.135 V, respectively [156].

Furthermore, the molybdenum-rich coatings also show high catalytic activity for the oxygen evolution reaction [157]. There are limited data on the catalytic activity of Fe-Re alloys for HER and OER or other electrochemical reactions, but these alloys show superior catalytic performance compared with monometallic Fe and Re catalysts in hydrogenation reactions [158].

Iron-based high-entropy alloys (HEAs) unlock new possibilities to control the functional properties of electrodeposits. For example, HEAs typically exhibit higher catalytic activity for HER compared with electrodeposited binary alloys. This is due to a larger number of active sites having different electronic structures and adsorption energies [159]. Commonly, the catalytic behavior of HEAs is associated with four core effects: a high entropy effect in thermodynamics [160], lattice distortion effect in structure [161], hysteresis diffusion effect in dynamics, and cocktail effect in properties [162].

Thus, due to the interaction of different elements and the high mixing entropy, the electrodeposited Ni-Fe-Cu-Co-W high-entropy alloys demonstrate a cauliflower-like structure and high catalytic activity. The excellent performance for OER can be attributed to several factors: (1) the synergistic effect among various metals enhances their intrinsic catalytic activity; (2) the binder-free self-supporting structure creates a shorter diffusion pathway for electrolyte penetration and facilitates faster electron transfer; and (3) the cauliflower structure increases the contact area between the electrode and the electrolyte. As a result, Ni-Fe-Cu-Co-W alloys, when electrodeposited at an optimal current density, achieve a lower overpotential value of 100 mV at 0.247 V, which is even smaller than that of many active OER catalysts such as IrO_2_ [163].

The synergetic effects observed in the electrodeposited FeCoNiMoW high-entropy alloy also have a significant impact on the magnetic and corrosion properties of these materials. Due to the unique microstructure of the deposited coatings and the relatively high content of non-magnetic elements (over 20 at.% of W and Mo), the coercivity (Hc) value is low, fluctuating between 11 and 18 Oe. Additionally, these alloys demonstrate excellent corrosion resistance, exhibiting a noble corrosion potential of *E_corr_* = −0.55 V and a low corrosion current density of *I_corr_* = 3.2 μA/cm^2^ [164].

Another possibility and advantage of exploring ferromagnetic alloys as catalysts is linked to the magnetic field application during the catalytic reaction. Thus, due to the synergistic effect of catalytic and magnetic action, it was possible to reduce by 10% the electricity needed for hydrogen generation compared with the base case scenario [165]. Moreover, the perspectives of iron-based magnetic materials as catalysts are not limited only to HER or OER reactions. For instance, the Fe-Ce-W magnetic catalyst can be smartly tuned to increase selectivity towards the reduction of various nitrogen oxides [166]. Additionally, assessing the stability of Fe-based catalysts is vital for their long-term application in practical engineering, particularly in oxidation processes [167], aggressive media [168,169,170], and fuel cells [171].

## 4. Summary and Future Perspectives

**Figure 5:**
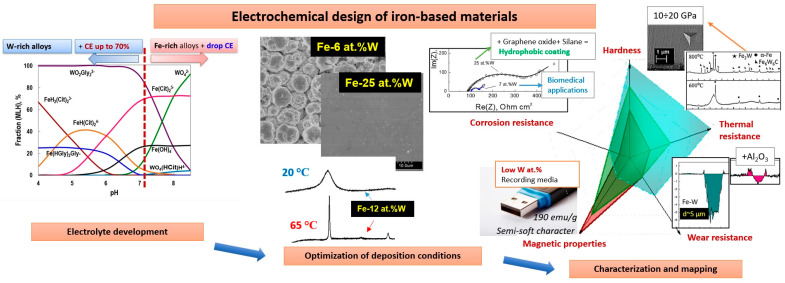
The outline of the electrochemical design of Fe-W alloys: from bath elaboration towards characterization and application.

We explored the potential of utilizing earth-abundant materials, specifically focusing on iron-based and refractory metal alloy electrodeposits. The properties of these materials can be easily adjusted through careful electrochemical design. For example, by simply shifting the pH of the solution, we can affect the distribution of complex species and obtain alloys that are rich in iron or tungsten with varying current efficiency (CE), as shown in Figure 5. This process leads to different compositions, morphologies, and structures, which can also be further fine-tuned by precisely controlling the temperature of the electrolytic baths. This control allows for transitions between nanocrystalline, ultra-nanocrystalline, or amorphous-like structures.

One of the main factors impacting the mechanical, magnetic, catalytic, and tribological properties of Fe-alloys is linked to the nanocrystalline structure of electrodeposited materials obtained from complexing solutions. Moreover, thermodynamic approaches can facilitate and optimize the electrosynthesis of nanocrystalline alloys. Namely, the knowledge of the complex speciation (Figure 5) in the solutions can help to: (a) define the compositional stability ranges of electrodepositing baths; (b) correct Pourbaix diagrams for particular conditions; (c) find the codeposition conditions and ways for process intensification; and (d) understand the influence of the composition of the bath on the composition and structure of electrodeposited alloys.

This interplay between parameters of electrochemical design is an opportunity to tailor such materials for versatile applications, which can be further enhanced by exploring the potential of micro- and nano-electrodeposits [172]. Namely, the excellent mechanical properties and thermal resistance of rich-W iron alloys can be explored for fusion reactors or other applications requiring thermal resistance. The soft magnetic Fe-rich alloys, along with their electrical properties, magnetostriction, and biocompatibility, can be explored for applications in micro-transformers, sensors, and NEMS fabrication. The mechanical properties and advanced biodegradability of iron-based scaffolds show potential for bone tissue engineering.

To adopt these alloys for tribological applications, the lubricated conditions should be applied to prevent the tribo-oxidation of iron-based alloys or to produce composites with added inert hard particles such as Al_2_O_3_, TiO_2_, or others. The alternative way to increase the mechanical robustness and wear resistance of electrodeposited materials is carburization, where the thermal decomposition of ethanol in an inert gas atmosphere at high temperatures is used to create carbide phases such as, e.g., Fe_3_W_3_C [173]. As we discussed earlier, these phases essentially contribute to increased hardness. Additionally, for targeted applications, the corrosion resistance of Fe-W alloys can be enhanced by increasing their hydrophobicity through the formation of silane cross-linked graphene oxide and hydrophobic organosilane layers on the base material (Figure 5). The catalytic properties can be tuned by the formation of nanostructured electrodeposits (e.g., Ni-Fe nanosheets) that can be rationalized afterwards by W doping [174]. Furthermore, electrodeposited NiFeW hydroxide electrocatalysts for efficient water oxidation can outperform conventional 20 wt.% Ir/C electrocatalysts [175].

Moreover, taking into account the growing focus on sustainability worldwide, the synthesis and discovery of new applications for earth-abundant materials will increase. However, iron-based alloys containing rare metals like Pt and palladium (Pd) demonstrate superior catalytic activity and stability compared with other alternatives. Their chemo-physical properties can be easily adjusted by altering the synthetic parameters. It is essential to choose the appropriate system, as the selection depends on the active phase needed for a specific reaction [176].

A sustainable method for obtaining the mentioned materials could involve conducting galvanic replacement reactions on iron or iron alloys using solutions that contain platinum or palladium derived from their waste-leaching processes. Thus, research has demonstrated the effectiveness of the galvanic replacement method for producing catalysts based on a Fe-Zn substrate [177]. However, substantial effort is still needed to fully understand and implement the eco-friendly electrochemical recovery of such materials.
